# The Study of Ætiology

**Published:** 1875-06-15

**Authors:** 


### Editorial Department.

### THE STUDY OF ÆTIOLOGY.

TO facilitate the study of ætiology, more especially in relation to acute endemic and epidemic diseases, we need much more exact knowledge in regard to the date of *commencement* of all cases occurring in a given number of places through a series of years.

Our mortuary statistics, showing the time and number of deaths in a given locality, do not supply the facts we need, for the well-known reason that death does not generally occur until several days and often weeks have elapsed after the disease began. Hence atmospheric conditions, that may have been potent in the production of an attack, may have ceased or become materially changed before the date of death. It is well known that systematic and detailed records of all important atmospheric conditions are now kept in localities representing all sections of our country, under the direction of the Signal Service Bureau of the General Government. And measures are being taken to render these records still more complete by adding observations on the electric and ozonic conditions of the atmosphere. If we could now secure the co-operation of a number of physicians engaged in active practice at their Signal Service Stations who would make it a duty to ascertain as exactly as possible the date of the first active symptoms of all acute cases of disease coming under their observation, entering the same in their pocket memorandums daily, and at the end of each month tabulate them on a sheet of paper properly ruled for that purpose, we would soon be able to determine whether there was any coincidence between certain atmospheric conditions and the development of certain diseases, by comparing the tables of sickness with those embodying the meteorological records in the same locality.

Initiatory steps were taken for securing such records by the Section on Practical Medicine of the American Medical Association at the meeting in Detroit, in 1874. The subject was further presented in a report to the same Section of the Association this year in Louisville, and a committee appointed to prosecute the work. We hope such practitioners as may be applied to for co-operation with the committee will cordially render their aid, and that each one will take all the necessary care to ascertain by careful inquiry as near the exact date of the initial symptoms in each case as is possible. The committee consists of Drs. N. S. Davis, and H. A. Johnson, of Chicago, and J. B. Johnson, of St. Louis.

